# Imidazole Headgroup Phospholipid Shows Asymmetric Distribution in Vesicles and Zinc-Dependent Esterase Activity

**DOI:** 10.3390/biom14111363

**Published:** 2024-10-26

**Authors:** Gabriela Sachet-Fernandez, James W. Hindley, Oscar Ces, Rüdiger Woscholski

**Affiliations:** 1 Department of Chemistry, Molecular Sciences Research Hub, Imperial College London, London W12 0BZ, UK; g.sachet-fernandez19@imperial.ac.uk (G.S.-F.); j.hindley14@imperial.ac.uk (J.W.H.); o.ces@imperial.ac.uk (O.C.); 2 Leverhulme Doctoral Scholarships Centre for Cellular Bionics, Molecular Sciences Research Hub, Imperial College London, London W12 0BZ, UK; 3 Institute of Chemical Biology, Molecular Sciences Research Hub, Imperial College London, London W12 0BZ, UK; 4 fabriCELL, Molecular Sciences Research Hub, Imperial College London, London W12 0BZ, UK

**Keywords:** phospholipase D, enzyme-assisted synthesis, allosteric, temperature, alcohol, lipid

## Abstract

Artificial lipids have become increasingly important in generating novel nanoenzymes and nanoparticles. Imidazole has been well established as a versatile catalyst in synthetic chemistry and through its related amino acid histidine in enzymes. By exploiting the transphosphatidylation reaction of phospholipase D, the choline headgroup of phosphatidyl choline was exchanged for the imidazole moiety containing histidinol. Here, we introduce a novel phosphatidylhistidinol (PtdHisOH) lipid and characterise it with respect to its catalytic abilities and its ability to modulate vesicle size. Our data reveal a zinc-dependent esterase activity that was strongest in vesicles and micelles, with slower catalytic rates being observed in flat lipid presentation systems and two-phase systems, indicating the importance of surface presentation and curvature effects on the catalytic activity of PtdHisOH. Such lipids offer the opportunity to impart de novo catalytic functionality to self-assembled lipid systems such as synthetic cells, leading to the development of new technologies for biocatalysis applications.

## 1. Introduction

The amino acid histidine is well known for forming an essential part of the catalytic site of many enzymes, which is mediated by the imidazole moiety [1]. Imidazole itself has also been employed as a catalyst in chemical synthesis, including nitro-aldol reactions [2]. Not surprisingly, it has also been the focus of many investigations to construct artificial enzymes, including ribonucleases [3], acylases [4], and esterases [5,6,7]. Many histidine enzymes sequester zinc as a cofactor to aid catalysis, and artificial enzymes mimic this approach [8,9]. Imidazole has also been employed to introduce pH sensitivity into liposomes and nanodelivery vehicles [10,11]. For this reason, the interaction of imidazole moieties with lipids has been investigated [12,13], but there is currently a void with respect to the catalytic properties of lipid vesicles containing imidazole headgroups.

Many of the artificial imidazole-containing lipids and liposomes made to date rely on organic synthesis for their generation [13,14,15]. More recently, artificial phospholipids were created by exploiting the transphosphatidylation reaction catalysed by the enzyme phospholipase D (PLD) derived from the microorganism Streptomyces, which exchanges the polar headgroup with suitable alcohol acceptor molecules [16]. However, so far, this method has not been employed to generate artificial lipids relevant for nanoenzymes and nanodelivery vehicles.

**Figure 1:**
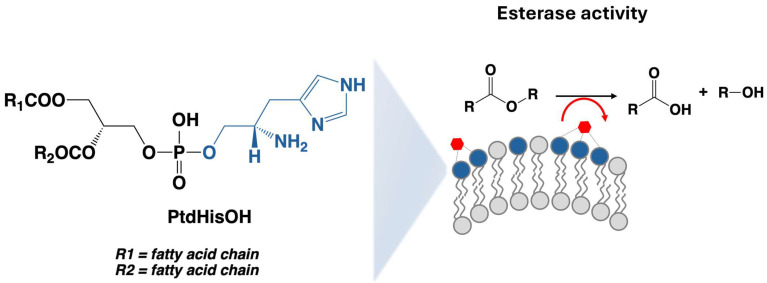
Catalytic properties of novel histidinol phospholipid presented in large unilamellar vesicles (LUVs). Phospholipids containing histidinol headgroups (blue circles) prefer to be located on the outside of the bilayer in unilamellar vesicles. They can bind zinc (red hexagons), and in this state, they catalyse ester hydrolysis (red arrow). The structure of PtdHisOH (shown on the left) reveals the imidazole and amine moieties (blue) present in the headgroup.

Here, we elucidate the catalytic properties of a novel imidazole-containing phospholipid synthesised using green chemistry principles by exploiting the PLD-mediated exchange of the choline headgroup in phosphatidylcholine (PC) with the natural imidazole alcohol, histidinol. The artificial lipid phosphatidylhistidinol (PtdHisOH) is characterised with respect to its effects on the architecture of membrane vesicles and its catalytic activity. We demonstrate that PtdHisOH lipid systems possess esterase activity that is dependent on the lipid being presented with the membrane of large unilamellar vesicles (LUVs) and is amplified by low zinc concentrations (see Figure 1). Our data imply that liposomal delivery systems featuring imidazole moieties will be affected by the intrinsic catalytic features of imidazole, and that PtdHisOH has the potential to be incorporated as a novel biocompatible catalyst that could find use as a component of lipid vesicle-based synthetic cell technologies [17].

## 2. Methods

### 2.1. Synthesis of Phosphatidylhistidinol (PtdHisOH)

#### Optimisation of Transphosphatidylation Reaction in Coated System

We recently established a plate reader assay to monitor the transphospatidylation reaction catalysed by PLD [18] that was adapted to optimise the synthesis of the histidinol phospholipid. Prior to the transphosphatidylation reaction, 10 μL of 10 mM DOPC (1,2-Dioleoyl-sn-glycero-3-phosphocholine) dissolved in CHCl_3_ was deposited into the wells of white 96-well plates (BRAND white polystyrene plate). Once the lipids are completely dry, a 50 μL solution containing 100 mM sodium acetate buffer at pH 5.6, 400 mM L-histidinol dihydrochloride (pH adjusted to 5.6), and 0.02 units of PLD (final concentration: 4 × 10^−4^ units/μL) from Streptomyces sp. (Type VII from Sigma-Aldrich, St. Louis, MO, USA) was added. After incubation (37 °C for 20 min), the plate was washed 3 times with sodium acetate buffer pH 5.6 followed by 3 washes with water. Variations in the conditions to this protocol are indicated in the appropriate figure legends.

### 2.2. Lipid Synthesis in Two-Phase System

**Figure 2:**
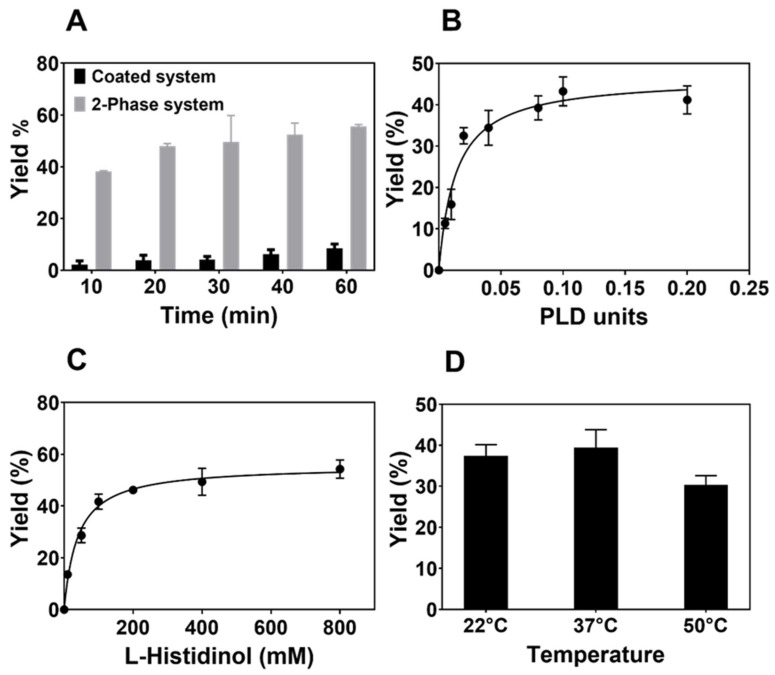
Optimisation of the transphosphatidylation reaction. (**A**) The coated versus two-phase system: In the coated system, DOPC (100 nmol) was deposited on the bottom of a 96-well polystyrene plate, while the two-phase system presented DOPC in CHCl_3_ (200 nmoles) in a plastic tube. The reaction was started by adding an aqueous solution containing L-histidinol (400 mM; pH 5.6), PLD (0.02 units), and BSA (0.05 mg/mL) and incubated at 37 °C for up to 60 min. (**B**) **PLD dose–response curve in the two-phase system:** the reaction was performed as described in part A with the aqueous phase containing the indicated amounts of PLD (up to 0.2 units) for 20 min. (**C**) **L-histidinol concentration dependency in the two-phase system:** the reaction was performed as described in part A with the aqueous phase containing the indicated amounts of histidinol (up to 800 mM) for 20 min. (**D**) **Temperature effect in the two-phase system:** The reaction was performed as described in part A with the aqueous phase containing the indicated temperatures for 20 min. Average yields (molar ratio of the PtdHisOH product versus PC starting material) from 6 experiments are shown, with the standard deviation being indicated as error bars.

The two-phase system, composed of an aqueous phase and a CHCl3 phase, was better suited for the synthesis of PtdHisOH, since higher yields can be obtained compared to the coated system (see Figure 2A). In general, a 50 mL round-bottom flask was employed for the reaction containing 10 mL of each phase. The aqueous phase contained 100 mM sodium acetate buffer pH 5.6, 400 mM L-histidinol dihydrochloride (pH adjusted to 5.6), 0.05 mg/mL of BSA, and 4 units of PLD from Streptomyces sp. (final concentration: 4 × 10^−4^ units/μL). The CHCl_3_ phase contained 2 mM DOPC. The flask was placed on a 37 °C hot plate, and the mixture is agitated using a stirrer bar for 40 min. The content of the flask was then centrifuged at 13,000× *g* for 3 min to aid separation of the two phases. The CHCl_3_ phases containing the lipids were stored at −20 °C.

#### 2.2.1. Monitoring the Transphosphatidylation Reaction

PtdHisOH can be detected either through thin-layer chromatography (TLC) or by fluorometric detection of the amino functionality present in PtdHisOH. For the TLC method, TLC aluminium sheets (silica gel 60) from Supelco were employed. A 2 μL volume of each standard (DOPC, 1,2-dioleoyl-sn-glycero-3-phosphate (DOPA), and 1,2-Dioleoyl-sn-glycero-3-phosphoethanolamine (DOPE)) and product was deposited on a plate. The TLC plate was placed into the chamber containing the mobile phase composed of chloroform/methanol/ammonium hydroxide (65:25:4 *v*/*v*), until it reaches the top of the plate. To visualise all phospholipids, the plate was stained with molybdenum blue spray reagent (Sigma-Aldrich). To visualise the amine group contained in PtdHisOH only, the plate was stained with ninhydrin (and then heated for 2 min at 100 °C to reveal the colour) or with fluorescamine (and exposed to UV light to reveal the fluorescence).

For the fluorometric method, lipids were deposited and dried in 96-well plates, and 50 μL of water is added to each well and topped up with 50 μL of 5 mM fluorescamine in acetone. The fluorescence was monitored in a plate reader (SpectraMax M3, Molecular devices, Wokingham, UK), at Ex/Em = 420/490 nm and at room temperature (~22 °C). The measurement was stopped once the reaction saturates and reaches a plateau, and the end point was measured.

#### 2.2.2. Purification of PtdHisOH

Column chromatography was employed for the purification of PtdHisOH, utilising silica gel 60 (0.063–0.200 mm from Millipore Sigma) as the stationary phase packed into a glass Pasteur pipette. The same eluent that was used for TLC, i.e., chloroform/methanol/ammonium hydroxide (65:25:4 *v*/*v*), was employed as the mobile phase. Fractions of 200 μL were collected, and their contents were verified using TLC as described above.

### 2.3. Production of Lipid Vesicles

Phospholipids were purchased from Cayman Chemical and Avanti, with PtdHisOH synthesised as described above. All lipids were stored in chloroform at −20 °C in a glass vial.

Lipids were mixed in CHCl_3_, dried under a nitrogen stream by desiccator exposure for a minimum of 4 h, and then employed to create LUVs and GUVs.

To make large unilamellar vesicles (LUVs) [19], the lipids were dispersed in HEPES buffer pH 7.4 to a final concentration of 5 mg/mL. The LUVs were prepared by extrusion, by passing the lipid dispersion 21 times through a 200 nm polycarbonate filter, using an Avanti Mini Extruder heated to ~65 °C.

To make giant unilamellar vesicles (GUVs) [20], the lipids were resuspended in mineral oil to a final concentration of 5 mg/mL of lipid and sonicated for 40 min at ~45 °C in a sonicator bath. GUVs were made by the emulsion phase transfer (EPT) using 0.5 M glucose and 0.5 M sucrose solutions. First, an emulsion was made using 20 µL of sucrose solution and 200 µL of lipid in oil. The emulsion was then deposited in a dropwise manner in an Eppendorf tube (1.5 mL) containing 300 µL of glucose solution. The tube was centrifuged at 9000 rcf for 40 min, and the GUVs were collected from the pellet and resuspended in fresh glucose solution. The GUVs were then added to the different assays as indicated in the methods below or figure legends.

To make micelles, lipid films were resuspended in HEPES buffer pH 7.4 containing 20 mM octyl-beta-glucoside. The glass vials were vortexed for 2 min and sonicated in a bath sonicator for 10 min.

### 2.4. Catalytic Activities of PtdHisOH

The esterase and phosphatase activities of PtdHisOH were analysed using artificial substrates: (i) FDA (fluorescein diacetate) for esterase activity and (ii) pNPP (p-nitrophenyl phosphate) for phosphatase activity (Refs [21,22]). Lipids were preincubated with divalent metals for 30 min prior to the start of the esterase or phosphatase assay.

For the esterase activity in the coated system, 10 nmol of lipid was incubated with or without 20 nmol of divalent metals (as indicated in the figure legend) in HEPES buffer pH 7.4 in the presence of 1 mM FDA. The reaction was monitored in a plate reader (SpectraMax M3, Molecular devices, Wokingham, UK), at Ex/Em = 480/520 nm and at room temperature (~22 °C) for 30 min. The same assay conditions were used when the lipid is presented in LUVs or micelles, using 20 nmol of lipid. In the two-phase system, the esterase reaction was performed in a 2 mL Eppendorf tube containing 100 µL of lipid in chloroform (20 nmol) and 100 µL of HEPES buffer pH 7.4 with or without divalent metals. Then, 1 mM FDA was added, and the aqueous phase was collected at 5, 10, 20, or 30 min after the initiation of the reaction. Fluorescence was measured in a plate reader as described above.

For the detection of the phosphatase activity, LUVs were incubated in a 96-well plate (clear) as described above, with or without divalent metals in HEPES buffer pH 7.4. After 30 min, 1 mM pNPP or 1 mM bNPP (bis-4-nitrophenyl-phosphate) was added to each well, and the plate was incubated for 30 min at 37 °C. The reaction was stopped by adding 10 µL of 750 mM NaOH to each well, and absorbance was read at 405 nm and at 600 nm.

Average catalytic rates were determined using the corresponding calibration curves (see Supplemental Figure S3). For each reaction, the slope was calculated from a 500 s time frame. Rates were calculated using the GraphPad Prism 9.2.0 software. The significance of errors was calculated with a standard *t*-test, with *p* > 0.1234 (ns), *p* > 0.0332 (*), *p* > 0.0021 (**), *p* > 0.0002 (***), and *p* < 0.0001 (****).

## 3. Results

### 3.1. Synthesis of PtdHisOH

Nanodelivery vehicles have been developed containing artificial lipids such as dilinoleylmethyl-4-dimethylaminobutyrate or 1,2-dimyristoyl-sn-glycerol, methoxypolyethylene glycol, and polyethylene glycol-dimyristoyl glycerol, but employ natural phospholipids such as PC as well [23]. To elucidate the role of imidazole in the lipid environment, we chose to synthesise a phospholipid with an imidazole headgroup that is as close as possible to the naturally occurring imidazole-containing compounds in the cella such as the amino acid histidine. This approach would facilitate the employment of the artificial phospholipid to be synthesised here in future investigations where strong cellular compatibilities are an important issue. The amino acid histidine and its metabolites generated in the catabolic or anabolic metabolism were obvious candidates. In particular, histidinol, a precursor in the biosynthesis of the amino acid histidine, seemed an ideal choice as it would allow one to utilise the PLD-mediated exchange of the headgroups of PC with histidinol. This reaction would exploit the efficient transphosphatidylation catalysed by PLD with primary alcohols [18]. While many investigations have explored transphosphatidylation reactions with a range of alcohols before, the synthesis of a histidinol phospholipid is without precedence. Thus, the optimal conditions for the enzyme-assisted synthesis were explored. We recently showed that multiwell plates could be employed to coat lipids on the bottom of the wells and investigate the transphosphatidylation reaction in a high-throughput format [18]. This system was compared to a two-phase system containing chloroform (see Methods for details) with respect to the yield generated. As can been seen in Figure 2A, the coated system delivered substantially lower yields as compared to the two-phase system. It is worth noting that the PLD enzyme is not just tolerating the chloroform in the two-phase system but is thriving under these conditions, which explains why this system is the method of choice when synthesising artificial lipids via the transphosphatidylation reaction [16]. The conditions for the synthesis were then further optimised with respect to the amounts of PLD and histidinol as well as the temperature in the two-phase system. The results obtained (Figure 2B–D) reveal that an efficient and consistent synthesis can be performed with 0.02 units of enzyme in the presence of 0.4 M histidinol in the aqueous phase at a temperature of 37 °C, with higher temperatures not being applied due to the low boiling point of chloroform facilitating evaporation, which in turn causes a higher error rate and reproducibility issues.

Employing the obtained optimised conditions for the two-phase system, the effect of scaling up the reactions (see Supplemental Table S1) was investigated. It is interesting to note that the drop in yields with larger reaction volumes could be compensated for by longer incubation times. The average yields were deemed to be acceptable for this study as artificial PtdHisOH would be presented in the experiments to follow in the presence of the carrier lipid PC. However, we also developed a method for the quick purification of PtdHisOH by removing the starting material DOPC with a simple silica-based chromatography step that elutes pure PtdHisOH in the unbound fraction, while retaining the PC on the column. The fractions of the column purification were analysed by TLC, revealing that the eluate is free of detectable amounts of PC and the possible by-product PA (see Supplemental Figure S1).

### 3.2. Catalytic Preference of PtdHisOH for Esterase Activity

Imidazole’s catalytic abilities are well characterised for the hydrolysis of carboxylic esters [8], but there is also evidence for the potential hydrolysis of phosphoesters [24]. To canvas whether the synthesised PtdHisOH is capable of cleaving any of the ester bonds, PtdHisOH/DOPC small unilamellar vesicles (LUVs) were employed in the first instance.

**Figure 3:**
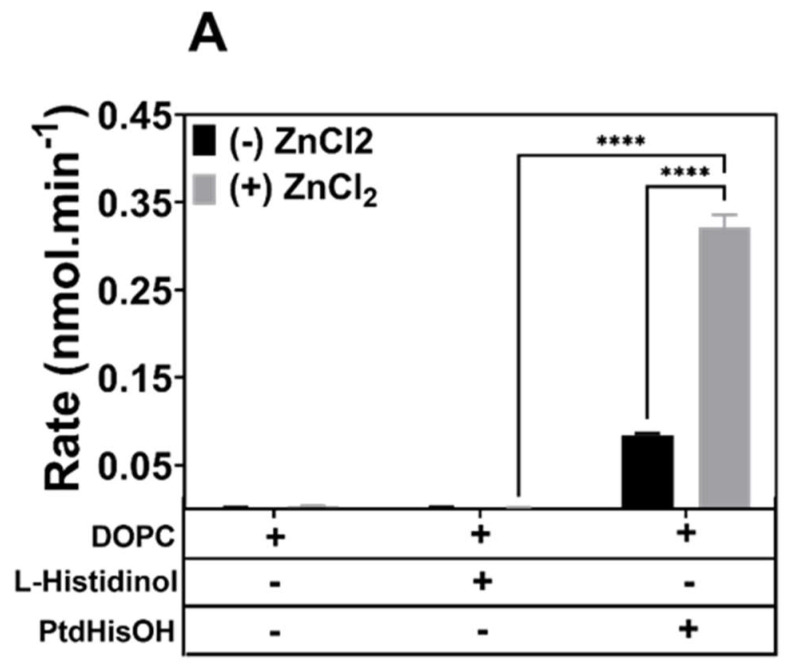

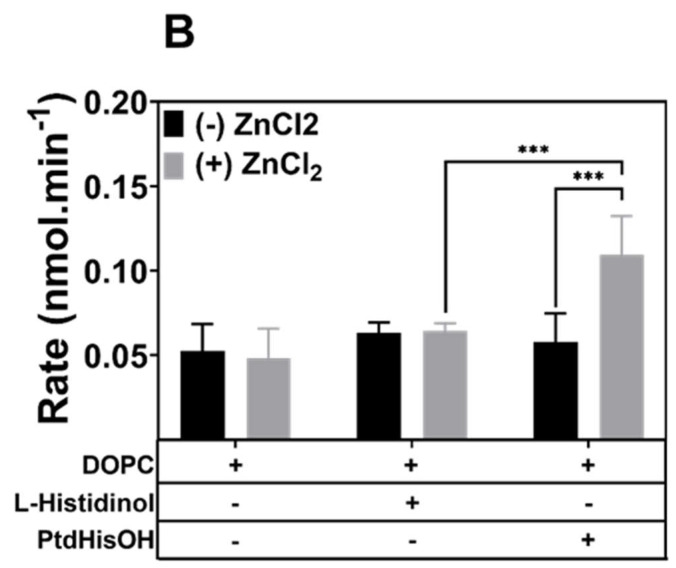
Zinc-dependent esterase and phosphatase activities of PtdHisOH. Esterase and phosphatase activities were tested for free L-histidinol (20 nmol) in the presence of DOPC LUVs and DOPC LUVs and for PtdHisOH (30 mol%)/DOPC LUVs. Lipids were preincubated with or without 20 nmol ZnCl_2_ for 30 min prior to monitoring the catalytic rates. (**A**) Catalytic rates for esterase activity: the reaction was monitored by the hydrolysis of 1mM FDA (Ex/Em = 480 nm/525 nm). (**B**) Catalytic rates for phosphatase activity: the hydrolysis of 1mM pNPP was measured after stopping the reaction (405 nm; 10 and 30 min). Average rates from 6 experiments are shown, with the standard deviation being indicated as error bars (see Section 2.4).

Since many catalytic activities in nature or in artificial settings employ zinc as a co-factor [8], we compared the activities in the presence and absence of zinc. As shown in Figure 3A, the presence of PtdHisOH in DOPC LUVs significantly increased the esterase activity (assessed via the hydrolysis of FDA) as compared to vesicles containing only PC or PC and the alcohol L-histidinol. Only the PtdHisOH/PC LUVs showed substantial esterase activity as well as an increased esterase activity in the presence of zinc. There was detectable phosphatase activity with PC vesicles and PtdHisOH/PC vesicles as assessed by the hydrolysis of pNPP (Figure 3B), but this was only significantly elevated in the presence of zinc for vesicles containing PtdHisOH. However, the esterase activity was boosted by 3-fold, whereas the phosphatase activity was boosted by less than 2-fold. Thus, the hydrolysis of esters seems to be the preferred catalytic reaction for this novel phospholipid. The addition of the alcohol L-histidinol to PC vesicles did not significantly boost esterase or phosphatase activity in the absence or presence of zinc. Binding zinc in natural enzymes requires at least three amino acids in close proximity, and for alpha carbonic anhydrases, these amino acids are all composed of histidines [25]. Thus, it is not surprising to observe a zinc dependency only in the PtdHisOH vesicles. The latter would facilitate zinc binding through the PtdHisOH density on the membrane surface of the vesicles, probably mimicking the necessary geometry for zinc binding, which the freely diffusible alcohol histidinol is unable to provide.

### 3.3. PtdHisOH-Dependent Esterase Activity Is Boosted by Zinc and the Presentation in LUVs

**Figure 4:**
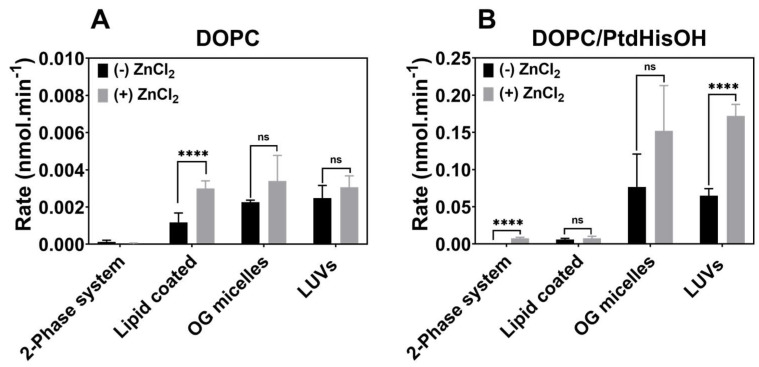
Effects of lipid presentation on zinc-dependent esterase activity. Lipids were presented either as LUVs or micelles (containing octylglucoside) coated on 96-well plates or in the two-phase system (buffer/CHCL3). DOPC was employed either alone (**A**) or with 30 mol% of PtdHisOH (**B**). Reactions were incubated with 1mM FDA, with (black bars) or without (grey bars) 20 nmoles of ZnCl_2_. The reaction (up to 30 min) was monitored by fluorescence and initial rates calculated using calibration standards (Supplementary Figure S3). Average rates from 6 experiments are shown for the different lipid presentations, except the two-phase system. For the latter, average rates from 4 experiments are shown. The standard deviation being indicated as error bars (see Section 2.4).

Having established that PtdHisOH in LUVs shows a zinc-dependent esterase activity, we investigated other lipid presentation systems. Lipids were presented either in mixed detergent (octylglucoside) micelles, coated on to a hydrophobic plastic surface (96-well plates), or were dispersed in the two-phase system used to synthesise PtdHisOH. As shown in Figure 4, the two-phase system was showing only detectable rates in the presence of zinc when PtdHisOH is present. The lipid coating did show a zinc-induced boost with DOPC but not with PtdHisOH/DOPC, but both showed low activities compared to the micelle and LUV presentation systems. Presenting the PtdHisOH lipid in micelles with octylglucoside or as LUVs showed good catalytic rates, which were boosted by zinc. However, only the LUV system showed a statistically significant boost by zinc (*p* < X), with the micelles having high error, and thus, no significant activity boost was observed in the presence of zinc.

The zinc-dependent catalysis boost therefore seems to be dependent on the different lipid presentation systems, with LUVs being better than other lipid presentation systems that will have different particle sizes and thus different surface curvatures. The two-phase system will have relatively large droplets in the µm range [26], with the LUVs being in the nm range [27], and the lowest particle size being attributed to mixed micelles [28]. As such, the curvature in these model systems will increase from the two-phase system (negligible curvature on the nanoscale) to mixed micelles (very high curvature). In contrast, in the coated lipid system, the phospholipids would be assembled as a flat bilayer with no curvature. It is interesting to note that the rates for PtdHisOH/DOPC (see Figure 4B) measured in the absence of zinc (black bars) are very low for the coated system and the two-phase system, whereas the micellar presentation and the LUVs show much higher rates in the absence of zinc, which in turn are higher than the equivalent rates for DOPC alone (see Figure 4A), indicating that the nanoscale curvature appears to have a positive effect on PtdHisOH catalysis.

The data presented in Figure 4 reveal a significant zinc-dependent catalytic activity in LUVs, which employed an equimolar ratio of zinc and PtdHisOH. We therefore investigated the effect of other bivalent metals (Co, Cu, and Ca) for their potential to replace zinc by comparing them to PE as a control, to account for the amino functionality and its possible contribution towards zinc binding. As shown in Supplemental Figure S2, only zinc boosted PtdHisOH- and DOPE-containing vesicles significantly, with zinc being by far the best metal. Neither cobalt, copper, nor calcium affected the rates in the same fashion as zinc when compared to the PC control LUVs.

Other metals that could interact with imidazole such as copper [29] or cobalt [30] did not show any significant metal-boosted esterase activities. For comparison, we also tested the effect of calcium, which has a similar charge to that of other metals, but has not been reported as having any esterase activity (see Supplemental Figure S2). These results also confirm that the esterase activity is caused by the zinc–imidazole interaction and not due to the bivalent-metal-induced changes in aggregations of the LUVs. In summary, our results indicate that PtdHisOH LUVs are strong zinc-dependent catalysts able to mimic esterase activity. To further investigate the zinc-dependent increase in catalytic rates in LUVs, the influence of the zinc/PtdHisOH molar ratio on the catalytic rates was investigated.

**Figure 5:**
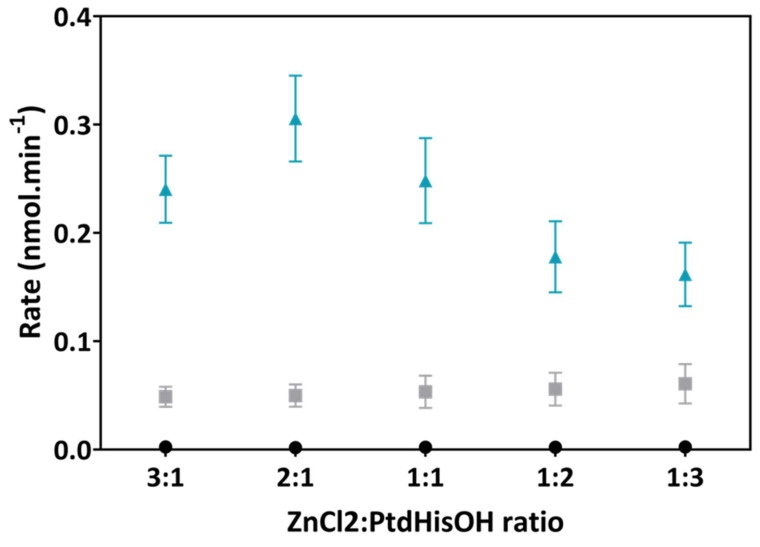
Optimal zinc/PtdHisOH ratios for esterase activities of lipids presented in LUVs. LUVs containing either 100 mol% DOPC (black dots), 70 mol% DOPC–30 mol% DOPE (grey squares), or 70 mol% DOPC–30 mol% PtdHisOH (cyan triangles) were incubated with increasing concentrations of ZnCl_2_ (keeping PtdHisOH at 20 nmol). Catalysis (30 min) and detection were carried out as detailed before. Average rates from 6 experiments are shown, with the standard deviation being indicated as error bars, against the zinc/PtdHisOH molar ratios.

The data presented in Figure 5 show that the optimal Zn^2+^/PtdHisOH ratio seems to be two zinc atoms per PtdHisOH lipid molecule in LUVs, with higher and lower ratios decreasing catalytic rates. No zinc dependency was observed for the control DOPC or DOPC/DOPE LUVs. At the lowest tested ratio of one zinc atom per three PtdHisOH molecules, about half of the optimum rate was observed. Given that the catalytic rate is dependent on the zinc metal, it is surprising that the drop in activity is only 2-fold, since the zinc concentration drops by 6-fold (2:1 versus 3:1 ratios). However, this would imply that three imidazole moieties are sufficient to chelate zinc and create a catalytically active complex, with an excess of zinc increasing the amount of this complex. This should result in a linear increase in the catalytic rate, which is not the case (6-fold increase in zinc leads to a 2-fold increase in activity). Therefore, it cannot be ruled out that the amino moieties in PtdHisOH contribute to the binding of zinc, which could explain that two PtdHisOH molecules are sufficient for forming an active complex and is observed to be an optimum ratio as per Figure 4. This complex could have a different catalytic rate to the one formed at a lower zinc concentration.

### 3.4. PtdHisOH Presented in LUVs Prefer the Outer Leaflet of the Membrane Bilayer

Since the LUVs are only able to form catalytic complexes with zinc on the outer layer of the LUVs, we investigated the distribution of PtdHisOH in the outer and inner layers of the LUVs. To investigate this possibility, the amount of free amino functionalities, present in PtdHisOH and PE, were determined in intact (in buffer) and dissolved (in 50% acetone) LUVs. Intact LUVs react with the amine-detection reagent fluorescamine only on the outer layer, while the acetone-treated LUVs dissolve and thus present all PtdHisOH lipids to the reagent.

**Figure 6:**
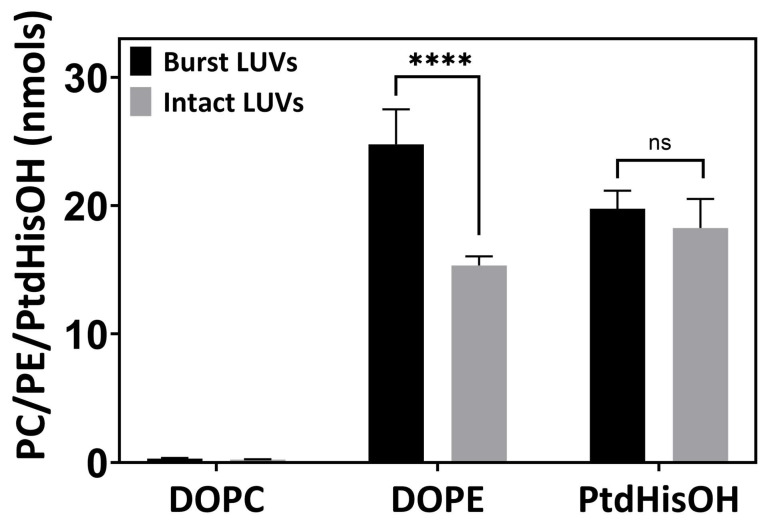
PtdHisOH distribution in the LUV bilayer. LUVs composed of 100 mol% PC, 70 mol% PC–30 mol% PE, or 70 mol% PC–30 mol% PtdHisOH were prepared by extrusion using a 200 nm filter. Each LUV sample (50 µL) in buffer pH 7.4 was supplemented with 50 µL of buffer containing fluorescamine, or 50 µL of acetone with fluorescamine, resulting in a final fluorescamine concentration of 2.5 mM. The kinetics of the reaction were monitored at Ex_420nm_/Em_480nm_, and the endpoints were recorded upon reaching saturation. Lipid quantities were calculated using the PE or PtdHisOH calibration curve with fluorescamine after treating LUVs with (black bars) or without (grey bars) acetone. Average rates from 9 experiments are shown, with the standard deviation being indicated as error bars (see Section 2.4).

As shown in Figure 6, LUVs containing either PE or PtdHisOH exhibited for PE a higher detectable amount in the presence of acetone as compared to the buffer control with intact LUVs. In contrast, the PtdHisOH LUVs did have almost equal amounts of amino functionalities in the LUVs with or without acetone, indicating that the LUVs have negligible amounts of amino functionalities inside the lumen of the LUVs. This, in turn, suggests that PtdHisOH is preferentially present on the outer leaflet of the LUVs, thus creating an asymmetric LUV membrane.

Given that the new imidazole headgroup of the phospholipid PtdHisOH had a strong preference for the outer leaflet of the LUV membrane, we could not exclude the possibility that the charges of zinc and imidazole might play a role in determining the sizes of LUVs.

**Figure 7:**
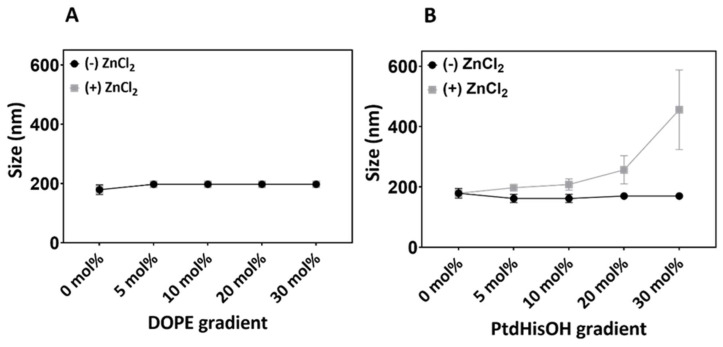
Particle size dynamics of LUVs with gradient PE or PtdHisOH. PE or PtdHisOH were presented with PC in LUVs using increasing amounts of lipid as indicated: (**A**) PC-PE LUVs (0–30 mol% PE) and (**B**) PC-PtdHisOH LUVs (0–30 mol%). LUVs were made by extrusion using 200 nm filters and were incubated for 30 min with (+) or without (-) 20 nmoles Zn^2+^. Particle sizes were measured using a particle sizer (Zetasizer) in a UV disposable cuvette. Data points in this graph represent the peaks for each size distribution curve generated. Average of 3 separate experiments (n = 3) and SD are shown.

To account for the possibility that the PtdHisOH density impacts LUV particle sizes, the size distribution of LUVs was analysed with increasing lipid molar ratios in the presence and absence of zinc. As can be seen in Figure 7, LUV sizes were not affected by either zinc and/or PE molar ratios. In a similar fashion, PtdHisOH molar ratios up to 30 mol% did not reveal any changes in particle sizes but were strongly increased in the presence of zinc at molar ratios of 20 mol% or above.

## 4. Conclusions

In summary, we have developed a new phospholipid containing a headgroup that is biocompatible and able to bind divalent cations. The new phospholipid PtdHisOH shows strong esterase activities in the presence of zinc, indicating a clear preference of zinc over other tested cations (Cu, Co, and Ca). The imidazole headgroup of PtdHisOH seems to be localised in the outer layer of the LUV membrane, showing a strong asymmetrical behaviour, but this seems to not affect particle sizes of LUVs, unless high mole percentages of PtdHisOH are employed. This new lipid will complement some of the existing lipid systems taking advantage of the imidazole properties, adding a strong lipid presentation-dependent catalytic ability that can be boosted by the presence of zinc. Embedding PtdHisOH in lipid membranes enables the generation of nano- and microparticles with esterase and pH-responsive functionalities. Such catalytic lipids could be easily integrated into membrane surfaces to generate catalysts with increased stability compared to enzymes or pH-sensitive functional groups that can improve the delivery of nanoformulations. Their asymmetric incorporation into the membrane surfaces also facilitates the creation of novel catalytic microenvironments that could be exploited in synthetic cell designs incorporating multiple spatial compartments [17]. As such, PtdHisOH lipids have the potential to be used in diverse applications from biocatalysis to the design of cell-like molecular assemblies.

## Data Availability

The original contributions presented in the study are included in the article/Supplementary Material, and further inquiries can be directed to the corresponding author.

## Supplementary Materials

The following supporting information can be downloaded at: https://www.mdpi.com/article/10.3390/biom14111363/s1, Figure S1: Thin Layer Chromatography of synthesised PtdHisOH; Figure S2: Metal dependency of PtdHisOH esterase activity in LUVs; Figure S3: Calibration curves for the detection of amino functionalities and esterase activities; Table S1: Optimization of the scale up: yields from reactions with increasing volumes.
