# Supplementary material for: Imidazole Headgroup Phospholipid Shows Asymmetric Distribution in Vesicles and Zinc-Dependent Esterase Activity
Source: Biomolecules. 2024 Oct 26;14(11):1363. doi: 10.3390/biom14111363 (PMC11592132; doi:10.3390/biom14111363)
Supplement: Supplementary file 1 [file biomolecules-14-01363-s001.zip › biomolecules-3217895-supplementary.pdf]

Supplemental information

Supplementary Table S1:

Optimization of the scale up: yields from reactions with increasing volumes

Yield %			Conditions		
Average yield	Std dev	n=	Vol aqueous phase	Vol organic phase	Reaction time
**47.9**	1.044	6	0.1 mL	0.1 mL	20min
**85.5**	9.697	6	1.5 mL	1.5 mL	20min
**65.7**	4.913	3	3.5 mL	3.5 mL	20min
**81.7**	5.695	6	10 mL	10 mL	40min

Supplementary Figure S1:

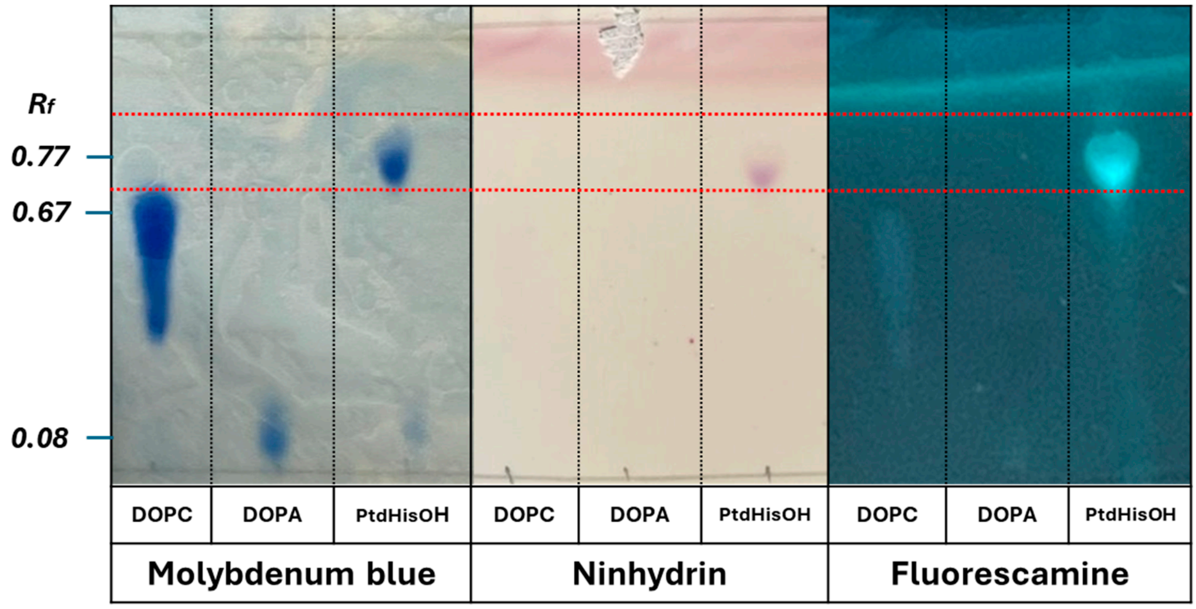

Thin Layer Chromatography of synthesised PtdHisOH

On a TLC plate, DOPC, DOPA (standards) and the $CHCl_3$ phase of the reaction (containing the synthesised PtdHisOH and the remaining starting material DOPC) were deposited. A mixture of CHCl3:MeOH:NH4OH (ratio 65:25:4) was used as the mobile phase. The plate was then stained either with Molybdenum blue (stains phospholipids), with Ninhydrin (stains primary amines) or with fluorescamine (reacts with primary amines). The Rf values for the corresponding lipid standards (DOPC=0.67;DOPA=0.08;PtdHisOH=0.77) are indicated at the left side of the images.

__Supplementary Figure S2: Metal selectivity__

Metal dependency of PtdHisOH esterase activity in LUVs.

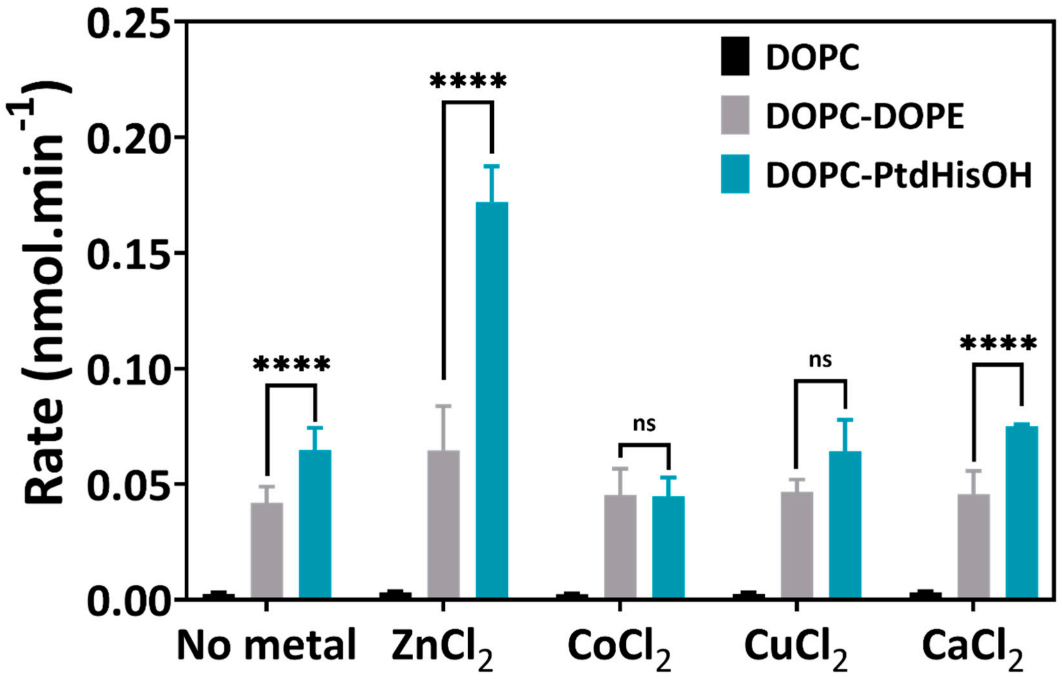

LUVs were created and esterase activity detected as specified in the methods section, with either no metal, or 20 nmoles of the indicated bivalent metal salts. Average of 2 separate experiments with triplicates (n = 6); Standard deviation being indicated as error bars (see section 2.4 of manuscript)

Supplementary figure S3:

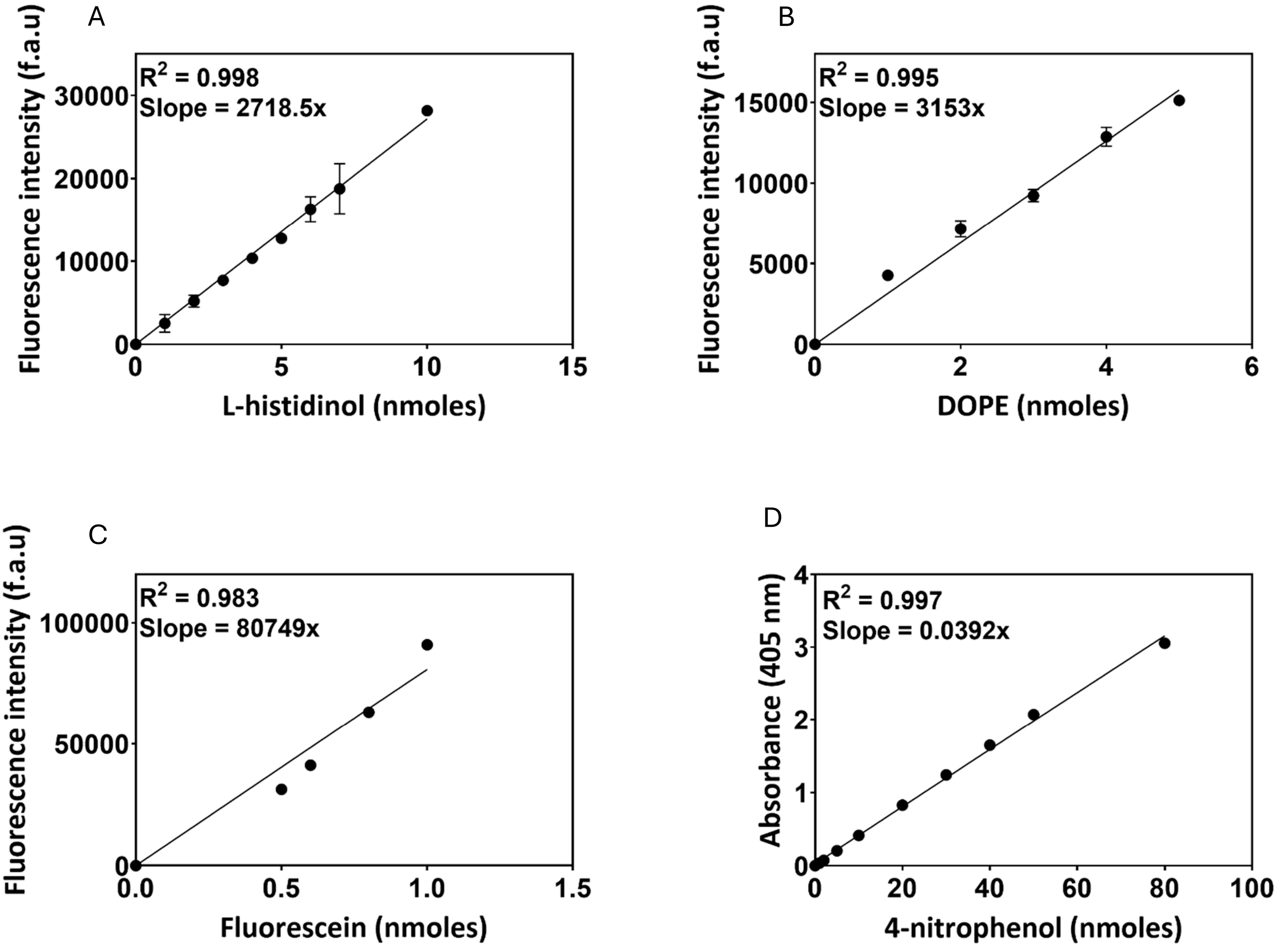

Calibration curves for the detection of amino functionalities and esterase activities.

Amino functionalities present in L-histidinol (A) and DOPE (B) were analysed in a white 96-well plate with 2.5 mM fluorescamine in a mixture composed of aqueous buffer and acetone (1:1 v/v) using the indicated amounts. The fluorescence of flourescamine was detected using a spectrometer at $Ex_{480\ nm}$ / $Em_{520\ nm}$. For the detection of the esterase activities with FDA the hydrolysed flourescein product was obtained by hydrolysing FDA using 0.25 units of esterase from porcine liver. Detection of the flourescein was performed in a spectrometer using $Ex_{420\ nm}$/ Em_{480nm} using the indicated amounts. With respect to the determination of the esterase activities with pNPP, the 4-nitrophenol

product was measured at 405 nm using a spectrometer in a clear 96-well plate, in alkaline conditions using the indicated amounts.
